# Numerical Simulation and ANN Prediction of Crack Problems within Corrosion Defects

**DOI:** 10.3390/ma17133237

**Published:** 2024-07-01

**Authors:** Meng Ren, Yanmei Zhang, Mu Fan, Zhongmin Xiao

**Affiliations:** 1 State Key Laboratory of Mechanics and Control for Aerospace Structures, College of Aerospace Engineering, Nanjing University of Aeronautics and Astronautics, Nanjing 210010, China; mrenmm@nuaa.edu.cn (M.R.); mfanz@nuaa.edu.cn (M.F.); 2 College of Aerospace Engineering, Chong Qing University, Chongqing 400044, China; zhangym@cqu.edu.cn; 3 School of Mechanical and Aerospace Engineering, Nanyang Technological University, Singapore 639673, Singapore

**Keywords:** buried pipeline, corrosion defect, crack tip, extended finite element method, neural network prediction

## Abstract

Buried pipelines are widely used, so it is necessary to analyze and study their fracture characteristics. The locations of corrosion defects on the pipe are more susceptible to fracture under the influence of internal pressure generated during material transportation. In the open literature, a large number of studies have been conducted on the failure pressure or residual strength of corroded pipelines. On this basis, this study conducts a fracture analysis on buried pipelines with corrosion areas under seismic loads. The extended finite element method was used to model and analyze the buried pipeline under seismic load, and it was found that the stress value at the crack tip was maximum when the circumferential angle of the crack was near 5° in the corrosion area. The changes in the stress field at the crack tip in the corrosion zone of the pipeline under different loads were compared. Based on the BP algorithm, a neural network model that can predict the stress field at the pipe crack tip is established. The neural network is trained using numerical model data, and a prediction model with a prediction error of less than 10% is constructed. The crack tip characteristics were further studied using the BP neural network model, and it was determined that the tip stress fluctuation range is between 450 MPa and 500 MPa. The neural network model is optimized based on the GA algorithm, which solves the problem of convergence difficulties and improves the prediction accuracy. According to the prediction results, it is found that when the internal pressure increases, the corrosion depth will significantly affect the crack tip stress field. The maximum error of the optimized neural network is 5.32%. The calculation data of the optimized neural network model were compared with the calculation data of other models, and it was determined that GA-BPNN has better adaptability in this research problem.

## 1. Introduction

The key to the technological development of long-distance gas pipelines lies in the balance between pipeline transportation efficiency and safety. In recent years, in the oil and gas transportation industry, in order to improve the transportation efficiency of pipelines, the operation of long-distance and high-pressure pipelines has become the norm. Under high internal pressure conditions, the risk of pipeline rupture will increase significantly. Especially when affected by other geological disasters such as earthquakes, the mechanical behavior of pipeline fractures will become more complex and dangerous. Earthquakes constitute a common natural disaster. Seismic waves propagate from the earthquake source to the ground, causing ground deformation. There are interactions between buried pipelines and surrounding soil, and pipelines will be damaged due to excessive deformation [1,2]. Detailed records of corrosion incidents are found in the “Metals Handbook” published by the American Society for Metals (ASM) [3]. For example, 65% of hazardous pipeline incidents between 1994 and 1999 were caused by corrosion in pipelines. There are various reasons for corrosion, mainly corrosion of pipeline transportation materials and corrosion of the soil environment. Buried pipeline networks will experience various complex soil environments such as alpine areas, undulating mountainous areas, and high-temperature areas, which greatly increases the risk of pipeline corrosion [4]. During the operation of long-distance pipelines, most of the environment is underground, making it difficult to monitor pipeline corrosion [5,6,7,8]. If the tiny cracks caused by corrosion cannot be discovered in time, they will not only have a great impact on the efficiency of oil and gas transportation but also cause damage to the soil environment around the pipeline, causing environmental pollution and soil erosion [7]. With the development of the social economy and the increasing demand for oil and natural gas, the reliability and safety analysis of buried pipelines has become more important.

In the published literature, a large number of studies have been conducted on the crack propagation or corrosion problems of buried pipelines. For example, Ariffin et al. studied the fracture response of multiple interacting cracks in pipelines under the action of large plastic strains and proposed a new strain-based CTOD estimation scheme [9]. Bin et al. used blasting experimental data to establish a failure pressure prediction formula for corroded pipelines in high-strength steel materials and verified it [10]. In order to predict crack initiation and crack propagation, the fracture toughness of pipes under internal pressure was studied [11]. Nykyforchyn et al. conducted an experimental study on the corrosion mechanical properties and hydrogen embrittlement behavior of intact X52 pipelines [12]. The extended finite element method is combined with experiments to simulate the damage parameter values required for crack propagation in the X52 pipeline [13]. The correlation between damage parameters and material yield strength and fracture toughness is discussed, and XFEM is used in Abaqus to simulate the crack propagation of the pipe [14]. Three-dimensional finite element analysis was used to study the crack growth behavior of repaired pipelines under cyclic internal pressure and crack growth modeling and remeshing were automatically handled by developing a parametric design language [15]. Compared with experimental methods, the use of numerical models to study cracked pipes can significantly reduce experimental costs, and the model calculation results are not very different from the experimental results.

In addition, the development of artificial neural networks and their applications in various major scientific fields provide new ideas for crack propagation and tip stress field prediction. Researchers have used the deep architectures of convolutional neural networks (CNNs) to detect cracks in structures without the need to compute defect features [16,17,18]. One of the more representative neural network models is the Back Propagation (BP) neural network. The BP neural network has an outstanding ability to solve nonlinear complex problems. It can be used to calculate cracks in road engineering maintenance, evaluate the condition of underground water pipes, and evaluate corrosion in buried pipelines [19,20,21,22]. In order to improve the accuracy of prediction, many researchers have optimized neural networks and obtained more applicable prediction models. For example, the Whale Optimization Algorithm (WOA) neural network hybrid model was used to study single-sided notched specimens, and standard parts were used for verification [23]. Other researchers combined the BP neural network and a genetic algorithm to predict the stress intensity factor (SIF) of ruptured pipelines [24]. An artificial neural network is trained based on a genetic algorithm to efficiently estimate the residual intensity of hydrogen damage, providing an important reference for corroded pipelines transporting hydrogen [25]. Based on the above research on cracks using neural networks, it is evident that BP neural networks exhibit excellent nonlinear mapping and generalization capabilities. Among commonly used neural networks, such as CNNs and RBF neural networks, BP neural networks are highly flexible and versatile, making them well-suited for a wide range of problems. Therefore, to address the issue of stress fields at crack tips in this study, the BP algorithm is highly suitable and can be considered the first-choice algorithm for the prediction model. This study uses the BP neural network to predict and analyze the crack tip stress field in the corrosion area of the buried pipeline and optimizes the BP neural network model based on a genetic algorithm. The prediction models are compared according to the calculated values of the finite element numerical model, and the better applicability of the prediction model under earthquake conditions is selected by means of the calculation error.

## 2. Physical Model

### 2.1. Extended Finite Element Model

For the purpose of fracture analysis in XFEM, the expansion function includes not only a near-tip asymptotic function that captures singular points around the crack tip but also a step discontinuity function that represents the displacement across the crack surface. The function used in the unit expansion partition to approximate the displacement vector function *u* is:

(1) $$u=\sum_{l=1}^{n}N_{I}(x)\left[u_{I}+H(x)a_{I}+\sum_{\alpha =1}^{4}F(x)b_{I}^{\alpha }\right]$$

There is a jump function when crossing the crack surface, which is a discontinuous step function crossing the crack surface. Formula (1) shows a sampling (Gaussian) point that represents the function of the point closest to the crack and the unit perpendicular to the crack vector. *F*(*x*) is a function showing the progressive crack tip in an isotropic elastic material. We used the crack tip function for isotropic elastic materials to account for the normal and tangential coordinates of the crack.

(2) $$F_{\alpha }(x)=\left[\sqrt{r}\sin\frac{\theta }{2},\sqrt{r}\cos\frac{\theta }{2},\sqrt{r}\sin\theta \sin\frac{\theta }{2},\sqrt{r}\sin\theta \cos\frac{\theta }{2}\right]$$

**Figure 1:**
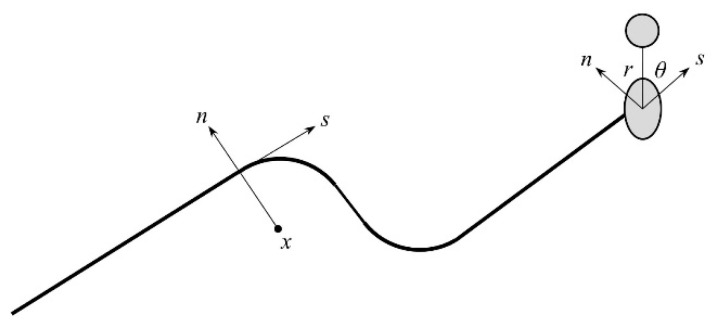
Schematic diagram of normal and tangential coordinates of smooth cracks.

Figure 1 is a systematic illustration of the polar coordinates on the crack tip. In Formula (2), r and θ are polar coordinate systems with the origin at the crack tip. When θ = 0, it means that the crack tip is tangential to the crack surface.

The extended finite element allows cracks to pass through the element. The grid and the section are independent of each other, so the discontinuous section needs to be geometrically described. The commonly used method is the level set method. The level set function is also used when constructing the extended shape function in XFEM.

**Figure 2:**
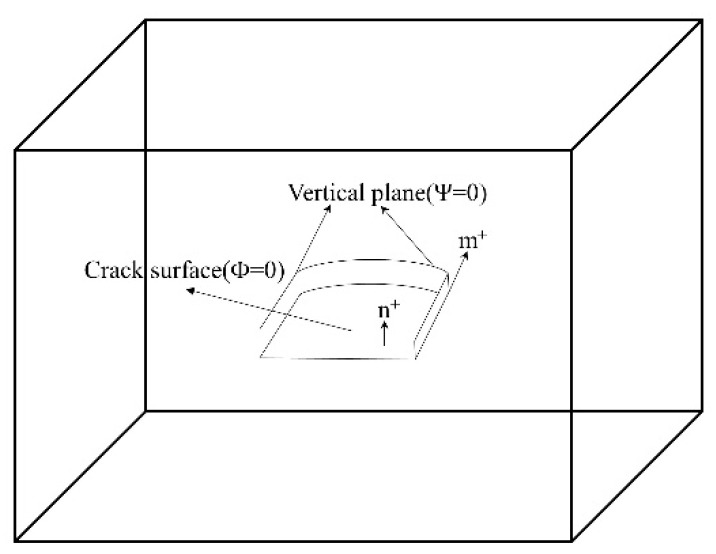
Distance functions ϕ and ψ for non-planar crack symbols.

**Figure 3:**
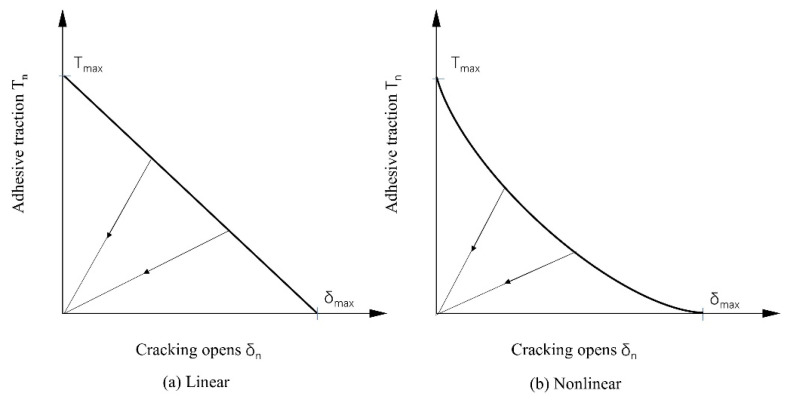
Typical traction-separation response [26].

The crack geometry is defined using two coincidence distance functions, as shown in Figure 2. The two intersecting surfaces define the crack front, and each node describes the specific crack geometry through these two signed distance functions. The failure mechanism of the crack unit in Abaqus includes two parts, one is the damage initial criterion and the other is the damage evolution law. After a damage initial criterion is met, the calculation can continue based on the defined damage evolution law. Figure 3 shows a typical linear and nonlinear traction-separation response.

**Figure 4:**
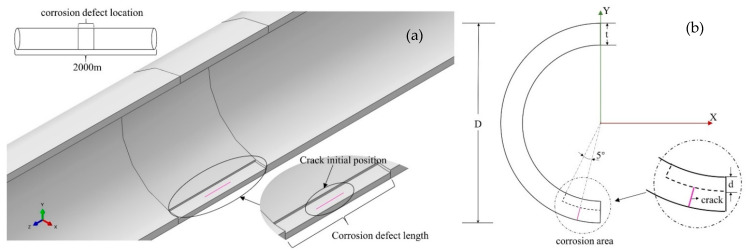
Crack location in corrosion defect, (**a**) the whole pipeline, (**b**) angle of crack distribution.

It is critical to model the areas of corrosion that occur. Research shows that local and uniform corrosion are the main forms of pipeline failure, and the corrosion area can be simplified into multiple forms during simulation modeling [27]. The shape of the corrosion area in the finite element analysis method is generally designed using a rectangle [28]. This study selects the most common uniform flat-bottom corrosion pit defects for analysis. As shown in Figure 4, setting the corrosion area below the pipeline and adopting symmetrical modeling will make the results more conservative. According to the B31G-1991 standard [29], the depth of corrosion defects should be greater than 10% of the nominal wall thickness and less than 80% of the nominal wall thickness. The defect depth is adjusted from 4 mm to 12 mm using a parametric method, and the depth range is controlled to 20% to 60% using this method.

**Figure 5:**
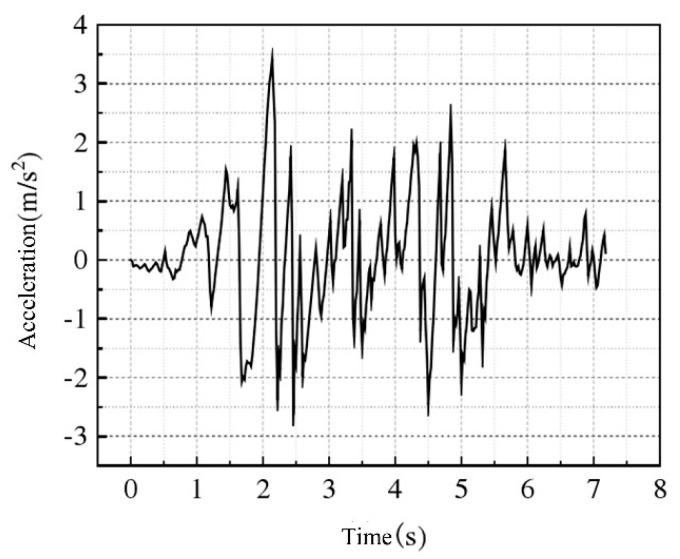
Seismic wave acceleration-time curve.

The incidence direction of the seismic wave propagating from the deep crust to the surface will gradually approach the vertical direction of the vertical–horizontal surface, and on this basis, the vertical incidence can be considered when the seismic wave input is carried out. In this study, the effect of vertical seismic waves on buried pipelines is analyzed by means of seismic wave input from the soil bottom. Seismic waves are selected according to site conditions, actual strong earthquake records, artificially simulated loads, and other reference points, and earthquake time history records suitable for buried pipelines are selected, as shown in Figure 5.

**Figure 6:**
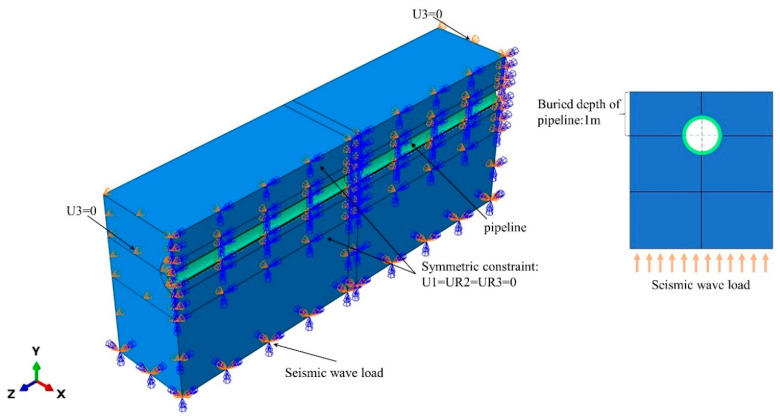
Modeling and simulation process.

Because both the pipe and soil are highly symmetrical structures, symmetrical modeling of the pipe and soil is carried out in order to save calculation costs and facilitate the observation of crack locations, as shown in Figure 6. The central position of the pipeline is 1 m away from the top of the soil model, and the simulated pipeline burial depth is 1 m. The soil mass is a cuboid with a length of 20 m, a width of 2 m, and a height of 4 m. A symmetric constraint in the X direction is established along the middle section Z, and the constraint is set as U1 = UR2 = UR3 = 0. The variable pressure is added inside the pipeline to simulate the internal pressure of the material transported in the pipeline, and the displacement constraint is applied to the bottom of the soil to simulate the seismic load as shown in Figure 6.

**Figure 7:**
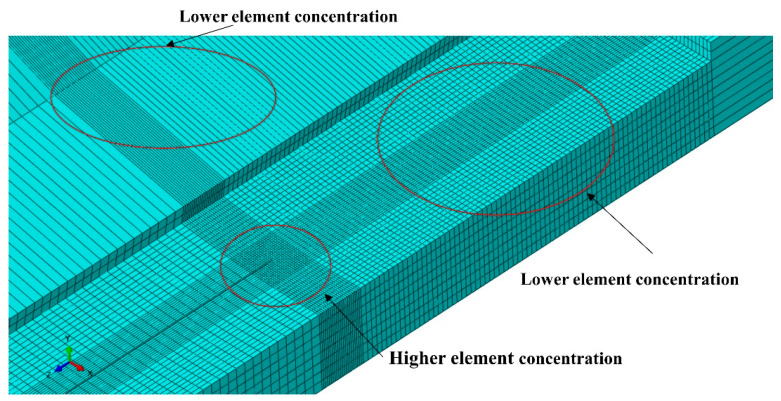
Corrosion defect meshing.

The model is built using C3D8R elements. Figure 7 shows the meshing method of the corroded pipeline model. The crack tip within the corrosion region was meshed with higher density as a key location for analysis. Hexahedral elements are used to divide the soil mass and the pipeline. The mesh of the contact part between the soil mass and the outer surface of the pipeline is encrypted, and the crack tip part is encrypted with a 10 mm square area. The mesh element is encrypted along the wall thickness direction on the pipeline. The minimum unit size of the pipe mesh at the crack tip was set as 0.5 × 0.5 × 0.5 mm^3^, and the incremental size of 0.5 mm was used to calculate the crack propagation length. Fewer grid cells are used at other locations in the pipeline in order to reduce calculation time. This approach also ensures calculation accuracy in the target area.

### 2.2. Neural Network Modeling

**Figure 8:**
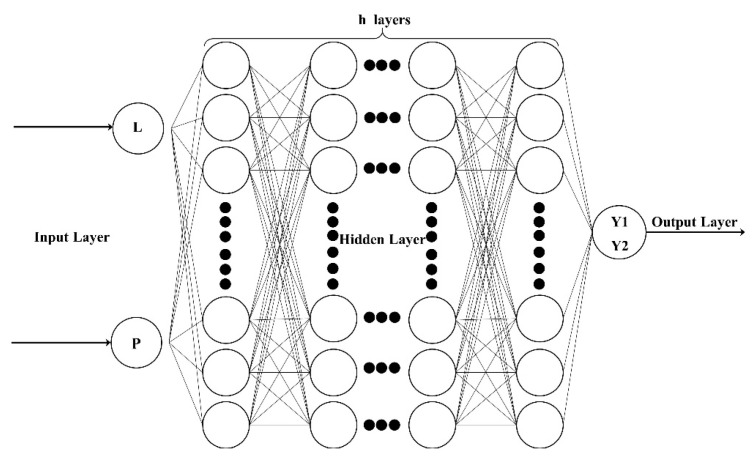
BP neural network structure diagram.

Parameters such as the number of hidden layer nodes, number of layers, and excitation functions in the BP neural network (BPNN) have a great impact on the prediction performance of the model. Currently, there is no definite method for selection [30,31,32], and the network structure parameters need to be designed. The input vector of the model is the initial length L of the crack and the change in the internal pressure P. The output vector is the stress Y1 at the crack tip and the crack expansion length Y2 calculated in XFEM. The initial model is shown in Figure 8. The parameters such as the diameter, wall thickness, and length of the pipe are set to constant values, the initial crack length L ranges from 2 cm to 20 cm, and the internal pressure P ranges from 1 MPa to 4 MPa. The input layer of the initial model has 2 nodes, the output layer has 2 nodes, the hidden layer node is set to m, and the number of layers is set to h. The values of m and h need to be determined based on experimental tests.

**Figure 9:**
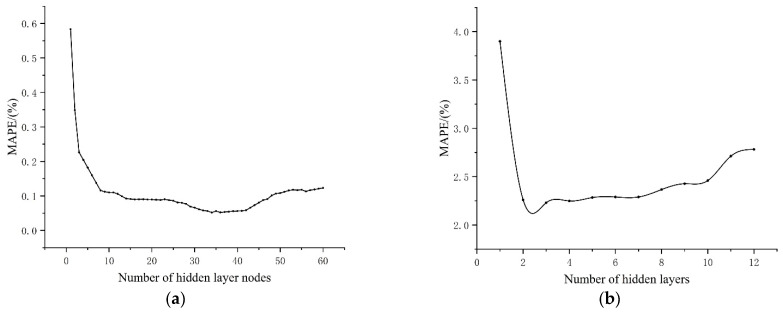
The relationship curve between MAPE and hidden layer. (**a**) Relationship curve between MAPE and hidden layer nodes. (**b**) Relationship curve between MAPE and number of hidden layers.

The approximate range of the number of hidden layer nodes can be determined based on relevant empirical formulas, but the precise value of the number of nodes cannot be determined and needs to be determined after multiple experimental tests and analyses. The mean absolute percentage error (MAPE) is analyzed as the experimental test result, as shown in Figure 9. Continuing to add hidden layer nodes will not increase the prediction accuracy. The appropriate number of hidden nodes should be selected by comprehensively considering issues such as training time. In this model, the number of hidden layer nodes is selected as 30, and the number of hidden layers is 2.

Based on experience, the excitation function at the hidden layer node in the BPNN model is selected as the tansig function, and the excitation function at the output layer is the logsig function. We nput 100 sets of test data into the BPNN model with set parameters and conducted experimental tests on the model, as shown in Table 1.

The minimum value of MAPE will appear when using the excitation functions purelin and tansig, and the model prediction effect at this time is the best. We set the BPNN hidden layer excitation function to the tansig function, and the output crack tip stress value will also be normalized to the interval [−1, 1]. This setting will also reduce part of the training time. The above completes the basic construction of BPNN. Next, the optimization of BPNN will be studied.

The initial weights and thresholds of BPNN are usually randomly generated, which can cause the sample to fall into a local optimal solution during the training process or cause problems such as slow convergence. The GA algorithm has stronger adaptability in solving global optimal solution problems and can adaptively optimize the search space and continuously optimize parameters such as weights and thresholds. These can help the BP algorithm get rid of over-reliance on gradient information and make up for the shortcomings of the BPNN model. The randomly selected weights and thresholds are extracted from BPNN and encoded in real numbers to form the chromosomes in the GA algorithm. We then completed the initialization of algorithm parameters, including population size, evolutionary algebra, crossover probability, mutation probability, etc. We then used the ga function to call the genetic algorithm for optimization, passing in the fitness function fitness and other parameters. The optimal weights and thresholds obtained by optimization are assigned to the BPNN, and the initial model of the BPNN optimized by the genetic algorithm (GA-BPNN) is obtained.

**Figure 10:**
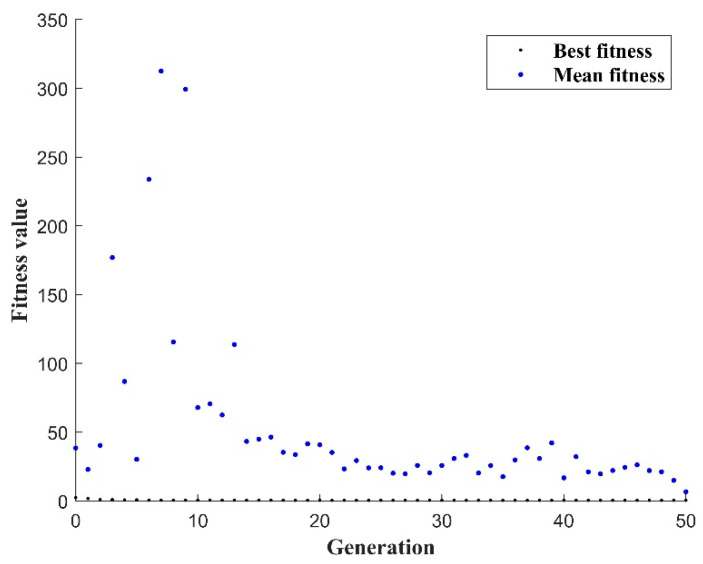
GA algorithm fitness function.

After 50 iterations in the GA-BPNN prediction model, the best fitness value and the average fitness value are not very different. The average fitness value tends to be stable. Although the optimal fitness value fluctuates slightly, it is basically stable within a certain range, as shown in Figure 10. The GA-BPNN model was constructed after 50 iterative calculations converged, proving that the previous parameter settings are reasonable.

## 3. Case Study

### 3.1. Crack Tip Stress Field Analysis

A numerical model was constructed in ABAQUS for analysis. First, the influence of the circumferential position of the crack on the tip stress field was studied. We controlled the angle of crack distribution change between 0° and 10° (from the edge of the corrosion zone to the bottom of the pipeline), set the corrosion depth on the pipeline to 4 mm, and used a combined load of seismic wave load and pipeline internal pressure. The internal pressure of the pipeline is set to a fixed value of 1 MPa. The data extracted through numerical simulation are used to analyze the influence of the distribution change in the axial crack in the circumferential direction on the stress field at the crack tip.

**Figure 11:**
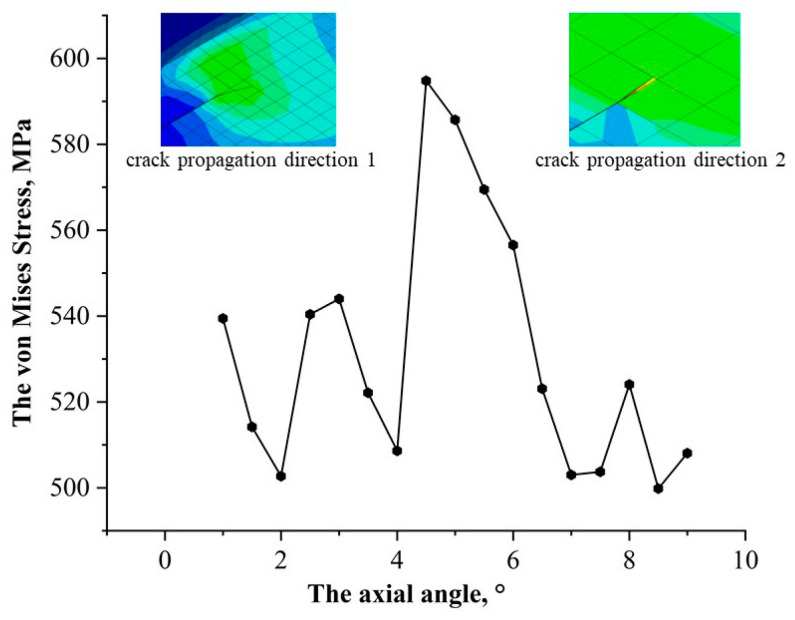
Relationship between crack circumferential distribution and crack tip stress.

As shown in Figure 11, the middle position of the corrosion zone is the position when the crack circumferential angle changes to approximately 5°. The equivalent stress value of the crack tip at this position will have a larger value. After deviating from this angle, the stress value at the crack tip begins to decrease. It can be seen from the crack propagation direction in the corrosion area in Figure 11 that after reaching the maximum stress value, the crack tip propagation direction also changes, and the range of stress fluctuations also begins to decrease. It can be seen from the solution results of the Abaqus numerical model that the expansion length that appears in the middle of the corrosion area is smaller than that in other locations. According to the energy fracture criterion, when the energy release within the crack is small, the crack driving force at the crack tip will have a larger value. The above shows that under the same load, the circumferential position of the crack in the buried pipeline will have a significant impact on the crack tip stress field.

**Figure 12:**
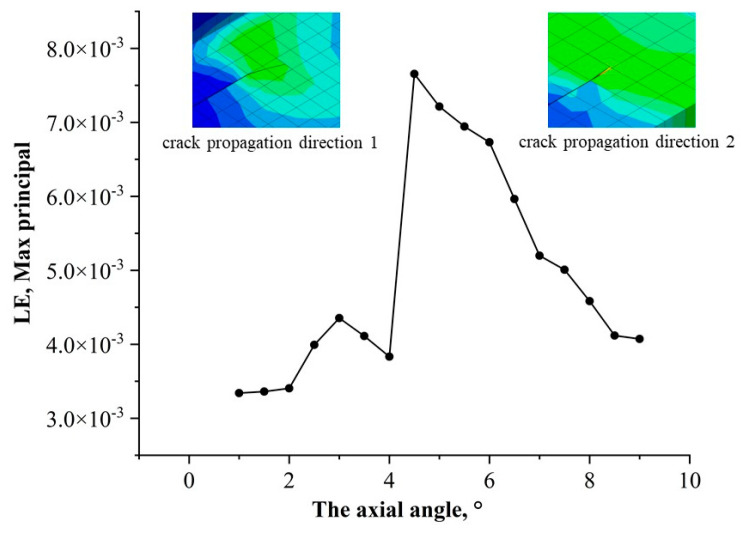
Relationship between crack circumferential distribution and crack tip strain.

The strain at the crack tip corresponds to the stress change, and the relationship between stress and the circumferential angle is shown in Figure 12. When the circumferential angle reaches around 5°, the strain value at the crack tip reaches a maximum value, and then the strain value continues to decrease as the angle increases. This further explains that cracks at different circumferential positions on buried pipelines will have different tip strains even under the same load, and the position has a great influence on the crack tip stress field.

Based on the above analysis, the crack in the middle of the corrosion area will generate greater stress. The stress field of the crack tip at different corrosion depths at this location was analyzed. The internal pressure of the pipeline varies between 1 MPa and 4 MPa, and the corrosion depth is 1 mm, 2 mm, 4 mm, 6 mm, 8 mm, and 10 mm. The crack tip characteristic curve is drawn based on the stress and strain data obtained from numerical simulation.

**Figure 13:**
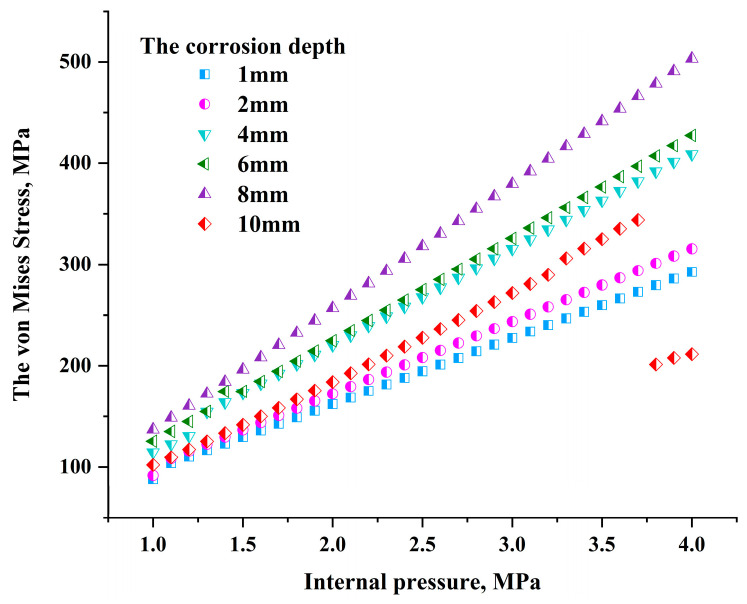
Relationship between crack tip stress and internal pressure under single load.

It can be seen from Figure 13 that under the action of only the internal pressure load of the pipeline, the stress value at the crack tip continues to increase. When the internal pressure of the pipeline changes between 1 MPa and 3.6 MPa, the crack tip stress at different corrosion depths shows an increasing trend. There is not much difference between the stress values at the crack tip of 4 mm and 6 mm depth. When the internal pressure of the pipeline is small, the crack tip in the corrosion area will have nearly coincident stress values, but when the corrosion depth increases to 8 mm, the stress will change significantly. As the internal pressure of the pipeline continues to increase, the increase in stress value at the crack tip with a corrosion depth of 8 mm is significantly greater than that of cracks with other depths, and the maximum stress value difference is approximately 100 MPa. When the depth reaches 10 mm, the corrosion degree of the pipe wall reaches 50%. When the internal pressure exceeds 3.6 MPa, the crack in the corrosion area expands and the stress value at the crack tip begins to decrease.

**Figure 14:**
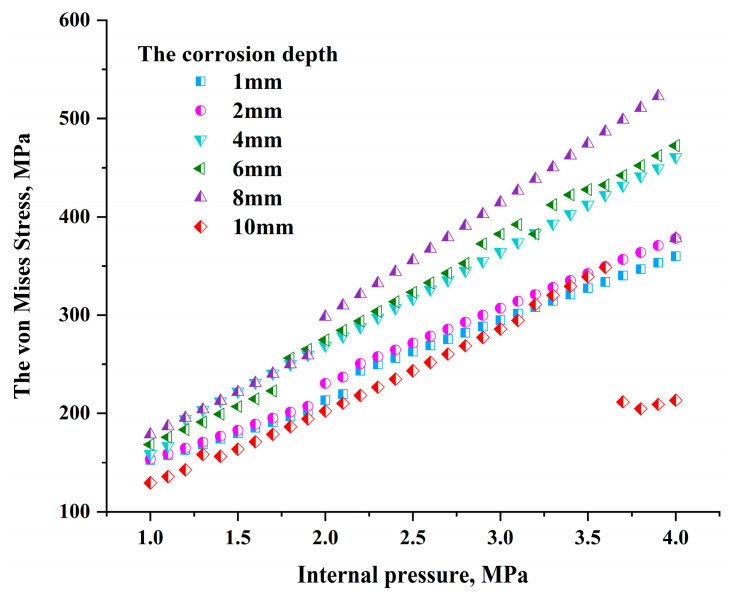
Relationship between crack tip stress and internal pressure under combined loads.

When the seismic wave load is added to the bottom of the soil model, the stress trend at the crack tip changes significantly. As shown in Figure 14, under the combined action of seismic wave load and pipeline internal pressure load, the difference in stress values at the crack tip at different corrosion depths on the pipeline becomes smaller. Compared with the case of only internal pressure load, when the depth reaches 8 mm, the pipe crack begins to expand, and the stress value at the crack tip fluctuates. Compared with a crack with a corrosion depth of 10 mm, the crack tip stress value with a corrosion depth of 8 mm is larger.

### 3.2. BPNN Prediction Analysis

First, the BPNN model is used to predict and analyze the crack tip stress field in the pipeline corrosion area. All the crack tip stress field data in the pipeline corrosion area calculated by Abaqus simulation are input as training samples, and the total number of training samples is 742 sets. Each sample is a four-dimensional vector, including pipeline corrosion depth, pipeline internal pressure value, crack circumferential angle, and crack tip stress value. The input vector corresponding to the BP neural network in each sample is a three-dimensional vector, and the output vector is a one-dimensional vector. The BPNN model we built is trained to obtain the crack tip stress field prediction model in the complete corrosion area.

In total, 180 sets of training samples are used to predict the crack tip stress field under multiple loads. The stress and strain data derived in finite element calculations are very different from the numerical magnitudes such as the pipeline corrosion depth, which will lead to gradient explosion problems and slow down the training speed. In order to avoid such problems, the crack tip stress field data need to be normalized. We used the mapminmax method to normalize the data to the interval [30,31,32] and input the normalized data into the BPNN model.

(3) $$y=(y_{\max}-y_{\min})\frac{X_{i}-X_{\min}}{X_{\max}-X_{\min}}+y_{\min}$$

y represents the value after normalization, $y_{\max}$ and $y_{\min}$ represent the maximum and minimum values of the data after normalization, respectively, $x_{\min}$ and $x_{\max}$ represent the maximum and minimum values before normalization, and $x_{i}$ represents the value before normalization.

**Figure 15:**
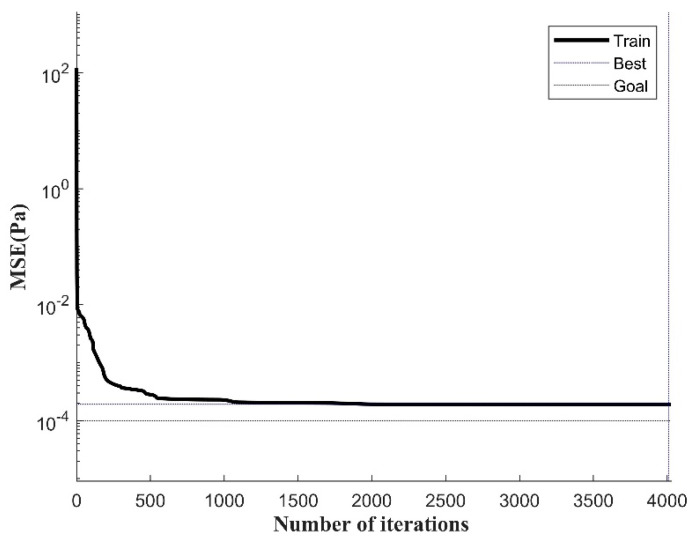
MSE curve during BPNN training process.

The mean square error (MSE) can intuitively determine the error between the predicted value and the true value. After model training, the training set data are exported through the saved neural network and error analysis is performed, as shown in Figure 15. The addition of seismic wave loads makes the stress changes at the crack tip in the corrosion area more complex. The BP neural network is prone to falling into local optimal solutions when dealing with nonlinear problems, making the model difficult to converge. In this case, the neural network model converges slowly and reaches the best training accuracy at 4027 iterations of training.

We used 180 sets of data to perform accurate error analysis on the BPNN prediction model. The calculation results in XFEM and the predicted results of BPNN were used to calculate the relative error, and some of the calculated results were randomly selected from all the calculated results and are organized in Table 2 below. Judging from the calculation error results, under the condition of the joint action of seismic waves and pipeline internal pressure, the prediction error of the BPNN model does not exceed 10%. The calculated average relative error is 0.72%, which meets the expected prediction requirements, but the training time is longer.

**Figure 16:**
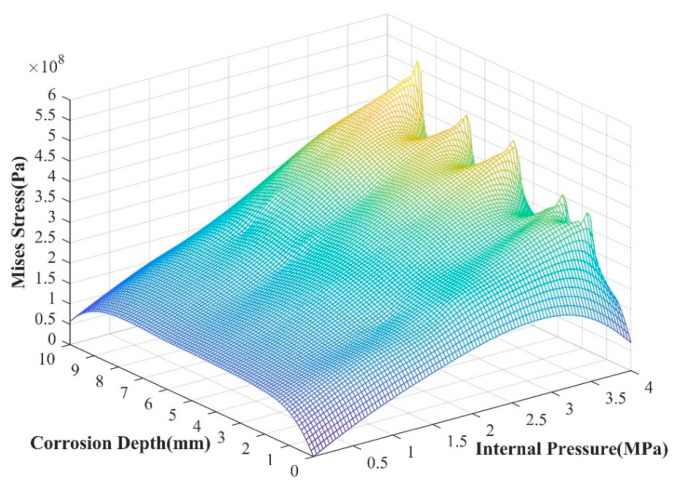
Crack tip stress changes with corrosion depth and pipeline internal pressure with BPNN prediction.

The input parameters are randomly selected within their respective value ranges to form sample data and introduced into the above model for prediction. The results are shown in Figure 16. As the corrosion depth increases, the difference in stress at the crack tip also changes. When the corrosion depth increases to 50%, large stress value fluctuations will occur, ranging from approximately 350 MPa to 500 MPa.

### 3.3. GA-BPNN Prediction Analysis

**Figure 17:**
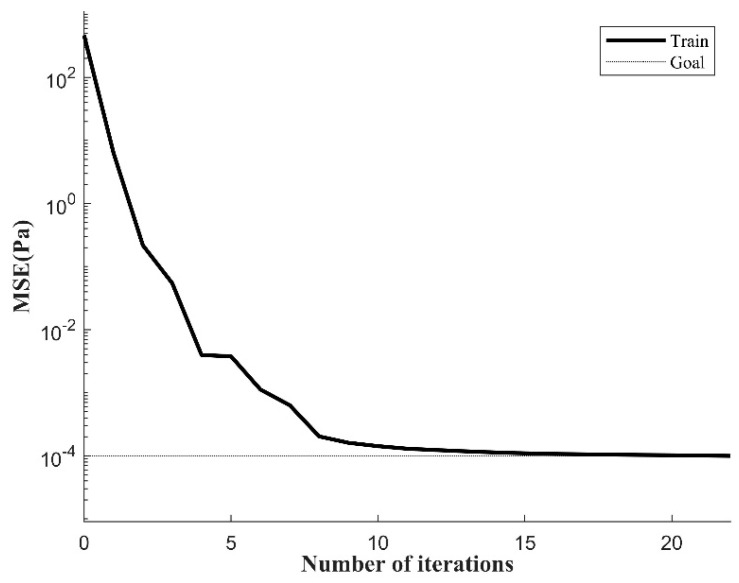
MSE curve during GA-BPNN training process.

The initial weight matrix of the BPNN model is optimized using the global optimization capability of the GA algorithm and the adaptability of the optimization object, which improves the accuracy of the BPNN model and reduces the training cost. The GA-BPNN is used to predict and analyze the crack tip stress field under the joint action of seismic waves and pipeline internal pressure loads. As shown in Figure 17, during the iterative training process, the convergence of the GA-BPNN is significantly better than that of the BPNN, and the training time is greatly shortened. After 15 iterative calculations of the training data, the model has converged. When the number of iterations is 22, the training accuracy is 0.0001, reaching the expected training goal. At this time, the model has converged.

An additional 180 sets of data were used to conduct prediction error analysis on the GA-BPNN model. The data of the input node are the depth of the pipeline corrosion area, the internal pressure of the pipeline, and the length of the crack, and the data of the output node are the calculated values of the crack tip stress.

**Figure 18:**
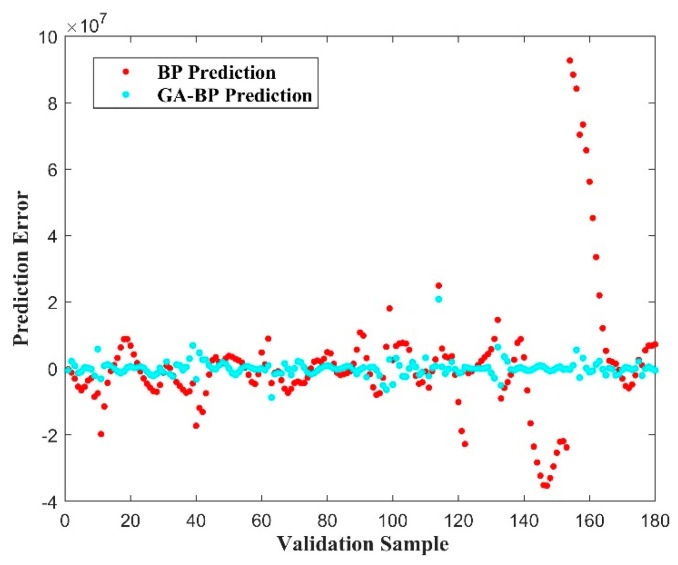
Comparison of calculation errors of prediction models for crack tip stress field.

Figure 18 shows the difference between the XFEM simulation calculation results and the GA-BPNN prediction results. Comparing the prediction results of BPNN, it can be found that the difference compared to the GA-BPNN is more concentrated near the 0 scale. In order to fully observe the prediction effect of the GA-BPNN model, the relative error of the prediction results is further calculated, and some calculation results are organized in Table 3. From the calculated error data, we can determine that the error in predicting crack tip stress using the GA-BPNN model is basically less than 6%, the average relative error is 0.57%, and the maximum error is 5.32%, which meets the basic requirements for pipeline engineering prediction.

**Figure 19:**
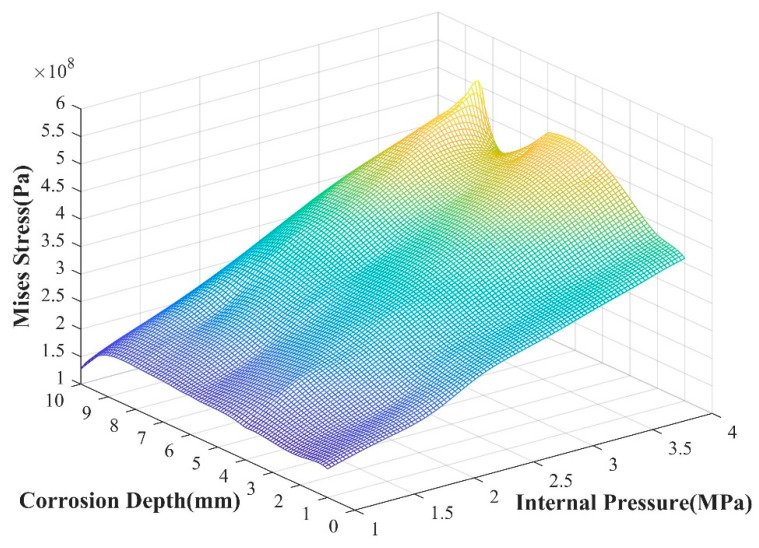
Crack tip stress changes with corrosion depth and pipeline internal pressure.

Parameters such as the depth of the corrosion area are randomly selected within their respective value ranges to form sample data, and these data are used as input parameters of the GA-BPNN model. We used the saved GA-BPNN prediction model to predict and analyze each data point of the internal pressure change under the action of seismic wave load. The results are shown in Figure 19.

Judging from the prediction results, when the internal pressure is 0, a stress value greater than 100 MPa appears at the crack tip. When the internal pressure of the pipeline is small, the stress value does not change much as the corrosion depth increases. When the internal pressure of the pipeline begins to increase, the stress value at the crack tip increases with varying amplitudes. The greater the corrosion depth, the more obvious the stress change trend at the crack tip. Due to the influence of seismic wave load, the stress value at the crack tip will fluctuate. This change is the same as the XFEM calculation result, which proves the accuracy of the GA-BPNN prediction model. Comparing the prediction results of the BPNN model, the numerical results of the optimized BPNN model for predicting the crack tip stress field under seismic wave loading are closer to the numerical calculation results, indicating that GA-BPNN is more accurate in predicting such working conditions.

### 3.4. Comparative Analysis of Prediction Models

In order to judge the applicability of the GA-BPNN prediction model to buried pipelines with cracks in other working conditions, it is necessary to conduct a comparative analysis on several other sets of data samples. The data under the action of only the internal pressure of the pipeline are recorded as sample A; the data under the combined action of the internal pressure of the pipeline and seismic wave load are recorded as sample B; the data of the corrosion-containing area under the action of internal pressure of the pipeline are recorded as sample C. Each sample consists of 100 sets of data. The longest training time and prediction results of the above data samples in different models are shown in Table 4.

We then randomly selected a set of data in sample B, used the XFEM model, BPNN model, and GA-BPNN model to perform calculation, and then compared their calculation times. The results are shown in Table 5. Judging from the time comparison of the three calculation models in the table, the neural network calculation time has obvious advantages, and the calculation results are basically consistent with XFEM. The calculation time of the BPNN model is approximately four times that of the GA-BPNN model. It can be seen that the optimized neural network has a great advantage in calculation time and greatly improves the efficiency of predicting the crack tip stress field.

**Figure 20:**
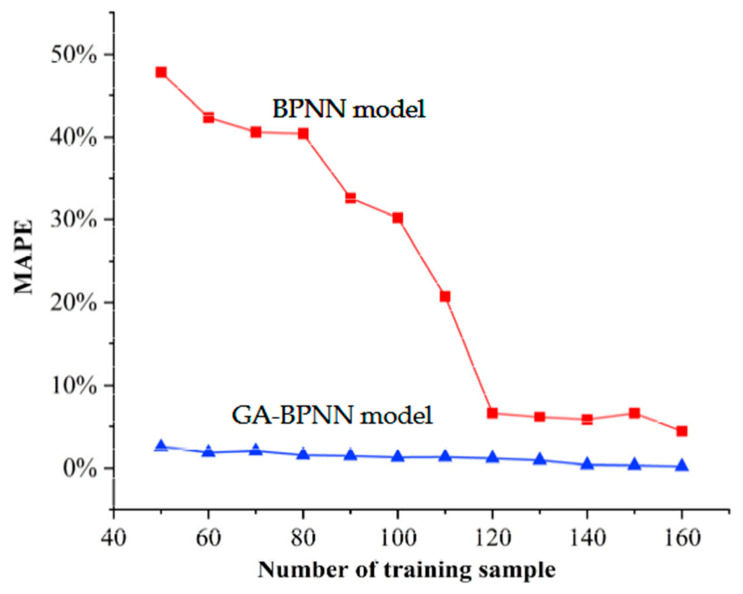
The impact of training data volume on prediction results.

We then divided the training samples in the above chapters into multiple groups of samples with different numbers and changed the amount of training data of the model to train the two prediction models. The obtained model was used to predict the pipe crack tip stress, and then its average absolute percentage error was calculated, as shown in Figure 20.

When there are only 50 sets of training data, the MAPE value of the BPNN reaches 50% and the prediction error is large. As the amount of training data increases, the prediction error of the BPNN model begins to decrease. It can be seen that the BPNN model has a strong dependence on the number of training samples. When using the same data samples to train the GA-BPNN, the prediction error of the GA-BPNN is basically maintained within 3% when the amount of data is small. As the amount of training data increases, the prediction error continues to shrink, indicating that the GA-BPNN model has low dependence on the amount of training sample data. In this study, the GA-BPNN has strong applicability for predicting the crack stress field of buried pipelines under variable loads.

## 4. Discussion

This paper uses the extended finite element method to establish a numerical model of buried pipelines and studies the stress field characteristics of the crack tip in the corrosion area of the pipeline under the action of multiple loads through parametric modeling. Based on the BP algorithm and the GA algorithm, a model that can predict the crack tip stress field in the corrosion area of buried pipelines is established. Compared with the existing research on buried pipelines [33,34,35], this model can accurately predict the crack tip stress field of buried pipelines under seismic load and can greatly reduce the calculation time. The main conclusions are as follows:

(1) Using single load and combined load as a comparison, it can be found that crack propagation is easier under the conditions of seismic wave load. When the initial length of the same crack in the pipeline is the same, there will be more crack propagation in the presence of seismic waves.
(2) The stress at the crack tip is affected by the depth of the corroded zone. When the corrosion depth is 20% to 30% of the pipe wall thickness, the crack tip stress value has little difference under the same load, and when the corrosion depth exceeds 40%, the difference in the crack tip stress value increases significantly. When the internal pressure value of the pipeline reaches 3.6 MPa, the stress value of the crack tip with a corrosion depth of 10 mm will decrease, and the crack will expand accordingly.
(3) The crack location in the corroded area also has an effect on the stress–strain at the tip. The maximum value of stress will occur when the crack is at a circumferential angle of 5°. At this position, the crack is located in the middle of the corrosion zone, and the crack propagation direction will change after passing this position.
(4) The BPNN prediction model is trained by using the training samples obtained from the numerical simulation data, and the prediction error of the neural network model is less than 10%. It is found that when the corrosion depth of buried pipelines under seismic load reaches 50%, the stress at the crack tip will fluctuate, with the fluctuation range being approximately between 450 MPa and 500 MPa.
(5) A GA-BPNN prediction model was established, which improved the prediction accuracy and significantly reduced model training costs. After further analysis of several sets of data samples, the training time, mean absolute percentage error value, and calculation time of the two models were compared, and the GA-BPNN was determined to have better adaptability.
(6) In future work, it can be considered how the interaction between pipe material and a corrosive environment affects the characteristics of the crack tip, using other neural network models [36] and comparing them with existing models [37], as well as the influence of a larger pipe internal pressure range and different seismic loads on the stress field at the crack tip, which can provide more comprehensive and detailed references for pipeline engineering.

## Figures and Tables

**Table 1:** Different excitation function MAPE values.

	Excitation Function 1	Purelin	Logsig	Tansig
Excitation Function 2				
purelin		62.084%	24.9305%	38.3906%
logsig		4.4202%	10.563%	4.8824%
tansig		2.0939%	5.7502%	10.7343%

**Table 2:** Analysis of crack tip stress prediction error.

Internal Pressure (MPa)	Corrosion Depth (mm)	Mises Stress (Pa)	Prediction (Pa)	Error (%)
1.00	4.00	158,858,240.7	158,780,917.5	0.05%
1.10	4.00	166,923,981.8	172,415,759.2	3.29%
1.20	4.00	194,278,477.3	187,248,842.9	3.62%
1.30	4.00	203,356,350	201,832,701.1	0.75%
1.40	6.00	199,002,554.2	197,161,845.5	0.92%
1.50	6.00	206,917,269.1	207,184,397.9	0.13%
2.00	6.00	274,833,262.7	275,189,147.5	0.13%
2.10	6.00	284,429,412	286,250,880.6	0.64%
2.20	6.00	294,036,021.3	295,401,659.5	0.46%
2.30	6.00	303,725,018.7	303,535,922.9	0.06%
2.40	8.00	344,150,725.3	346,682,699.9	0.74%
2.50	8.00	355,773,033.3	357,428,946.5	0.47%
3.10	8.00	426,593,936	425,214,183.8	0.32%
3.20	8.00	438,539,185.3	438,319,073.8	0.05%
3.30	8.00	450,516,069.3	452,357,812.2	0.41%
3.40	8.00	462,521,981.3	465,606,716.2	0.67%
2.80	10.00	268,782,703.1	270,518,563.7	0.65%
2.90	10.00	277,353,961.2	277,740,658.4	0.14%
3.00	10.00	285,967,145.8	285,472,489.9	0.17%
3.10	10.00	294,618,943.4	295,278,673.3	0.22%
3.20	10.00	310,792,703.1	307,250,193.3	1.14%
3.30	10.00	320,085,231.1	319,514,358.9	0.18%

**Table 3:** The predictions and errors of the ANN model with a single input volume.

Internal Pressure (MPa)	Corrosion Depth (mm)	Mises Stress (Pa)	Prediction (Pa)	Error (%)
2.10	4.00	297,548,268	298,136,897.6	0.20%
2.20	4.00	307,077,117.3	309,945,362.9	0.93%
2.30	4.00	316,619,324	319,584,861	0.94%
2.40	4.00	326,172,602.7	327,029,224.3	0.26%
2.50	4.00	335,741,713.3	334,648,014.3	0.33%
2.60	4.00	345,309,794.7	344,315,836.5	0.29%
2.10	6.00	303,725,018.7	302,351,752.3	0.45%
2.20	6.00	313,453,465.3	313,573,244.5	0.04%
2.30	6.00	323,217,749.3	323,932,440.5	0.22%
2.40	6.00	333,014,658.7	332,830,976	0.06%
2.50	6.00	342,841,344	342,625,170.5	0.06%
2.60	6.00	352,695,277.3	355,128,398.2	0.69%
2.10	8.00	332,594,832	333,493,727.7	0.27%
2.20	8.00	3441,50,725.3	343,971,431.9	0.05%
2.30	8.00	355,773,033.3	356,102,716.5	0.09%
2.40	8.00	367,455,537.3	367,702,048	0.07%
2.50	8.00	379,193,017.3	378,137,007.4	0.28%
2.60	8.00	39,0979,374.7	389,107,384.8	0.48%
2.10	10.00	226,702,972.9	228,260,331.6	0.69%
2.20	10.00	234,998,611.4	234,972,060.8	0.01%
2.30	10.00	243,360,038.2	241,860,620.5	0.62%
2.40	10.00	251,781,316	251,142,109.8	0.25%
2.50	10.00	260,257,083.7	261,156,249.2	0.35%
2.60	10.00	268,782,703.1	269,412,972.3	0.23%

**Table 4:** Training time and MAPE value before and after artificial neural network optimization.

Model	Training Time (s)	MAPE of Sample A (%)	MAPE of Sample B (%)	MAPE of Sample C (%)
BPNN	30	0.6501%	12.2363%	0.67934%
GA-BPNN	11	0.8044%	1.7560%	0.25615%

**Table 5:** Computation time for different models.

Model	XFEM	BPNN	GA-BPNN
computation time	18 min	28 ms	7 ms

## Data Availability

The raw data supporting the conclusions of this article will be made available by the authors on request.
